# A Sensorimotor Model for Computing Intended Reach Trajectories

**DOI:** 10.1371/journal.pcbi.1004734

**Published:** 2016-03-17

**Authors:** Cevat Üstün

**Affiliations:** Division of Biology, California Institute of Technology, Pasadena, California, United States of America; Johns Hopkins University, UNITED STATES

## Abstract

The presumed role of the primate sensorimotor system is to transform reach targets from retinotopic to joint coordinates for producing motor output. However, the interpretation of neurophysiological data within this framework is ambiguous, and has led to the view that the underlying neural computation may lack a well-defined structure. Here, I consider a model of sensorimotor computation in which temporal as well as spatial transformations generate representations of desired limb trajectories, in visual coordinates. This computation is suggested by behavioral experiments, and its modular implementation makes predictions that are consistent with those observed in monkey posterior parietal cortex (PPC). In particular, the model provides a simple explanation for why PPC encodes reach targets in reference frames intermediate between the eye and hand, and further explains why these reference frames shift during movement. Representations in PPC are thus consistent with the orderly processing of information, provided we adopt the view that sensorimotor computation manipulates desired movement trajectories, and not desired movement endpoints.

## Author Summary

Does the brain explicitly plan entire movement trajectories or are these emergent properties of motor control? Although behavioral studies support the notion of trajectory planning for visually guided reaches, a neurobiologically plausible mechanism for this observation has been lacking. I discuss a model that generates representations of desired reach trajectories (i.e., paths and speed profiles) for point-to-point reaches. I show that the predictions of this model closely resemble the population responses of neurons in posterior parietal cortex, a visuomotor planning area of the monkey brain. Several aspects of population responses that are puzzling from the point of view of traditional sensorimotor models are coherently explained by this mechanism.

## Introduction

How do we reach to what we see? The answer to this sensorimotor problem may seem evident as we prepare to grab a cup in front of us. The location of the cup is first determined within visual coordinates, but motor commands need to be specified with respect to the arm. A reasonable assumption is that to produce movement, the brain needs to convert goal representations between these two coordinate frames, and that transitional goal representations during this process should be expressed with respect to readily identifiable, intervening parts of the body, such as the head, the body, the shoulder, and so on [1]. Surprisingly however, recordings of neural activity from sensorimotor areas show that goals are encoded in reference frames that span the continuum between such intuitive cases. For instance, in reach-related areas of posterior parietal cortex (PPC), a neuron may encode reach goals with respect to the eye, with respect to the hand, or with respect to an arbitrary point in between these two [2–4]. Similar results have been reported for sensorimotor modalities in the ventral [5] and lateral [6] intraparietal regions, parietoinsular vestibular cortex [7], and superior colliculus [8].

The origin of these functionally intermediate representations remains unclear. An appealing explanation is that they represent units whose responses have been corrupted by noise. Recent studies have shown this to be an unlikely explanation for reach-related neurons in PPC, however, and have suggested that units with intermediate reference frame encodings may serve a distinct, albeit currently unidentified, role [2]. It might then be expected that simulations of the presumed sensorimotor computations (i.e., coordinate shifts) using neural networks could yield insight into what this role might be, yet such studies have yielded only partial answers. While certain features of sensorimotor activity (such as gain modulated tuning) arise naturally from backpropagation-trained networks, intermediate representations do not necessarily follow [9–11]. An exception to this occurs in the case of networks constructed to perform sensorimotor integration using both sensory-to-motor and motor-to-sensory computations [12,13]. However, recent single-cell studies suggest that the anatomical organization of cortical representations differs from the topographical predictions of these models [14]. A further complication is that in PPC, reference frames (including functionally intermediate ones) are found to have dynamic properties: encoding frames for reach targets are stable immediately before movement onset [15], but they transition towards an eye-centered frame during movement [4,16]. Given the accumulation of these findings, we may question whether the representations underlying our actions are indeed stable and systematic [2,17]. Alternatively, we may ask if the assumptions of the models used in interpreting the data are appropriate.

There is in fact compelling behavioral evidence to suggest that traditional sensorimotor frameworks need revision, at least in the case of goal directed reaching. A core assumption of such models is that sensorimotor computation involves the representations and transformations of goal locations only [1,18–22]. An implication is that the planning of reach trajectories (i.e., paths and speed profiles) must then be carried out within the late stages motor control, and the resulting reaches should therefore show evidence of being planned in intrinsic coordinates close to motor output, such as in joint angles. Yet behavioral experiments consistently show that extrinsic (Cartesian) coordinates provide a better description of reach trajectories than joint angles [23–25]. The relative straightness of movement paths in extrinsic space is a striking feature of such experiments, and the residual curvatures in the movement paths have been interpreted as evidence for the planning of movement in visual coordinates [26,27].

**Fig 1:**
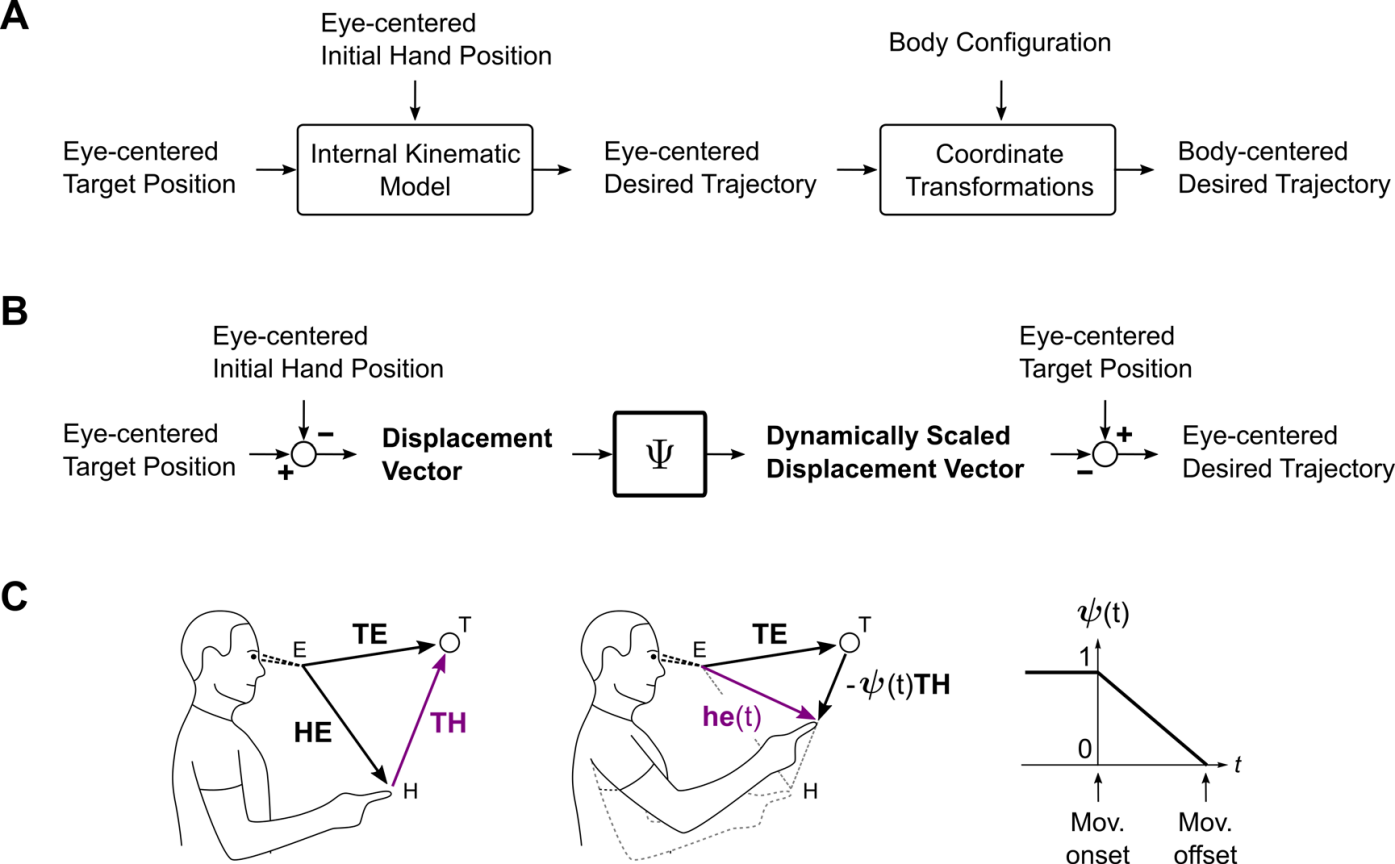
Sensorimotor transformations and the internal kinematic model. (A) Sensorimotor model for visually guided reaching. A module, termed an internal kinematic model, generates a desired reach trajectory, in visual coordinates. Movements can then be executed in visual space, even though motor commands are ultimately specified in intrinsic (body-centric) coordinates. Sensory feedback loops and associated mechanisms are assumed, but omitted. (B) Computations required for an internal kinematic model. Temporal and spatial operations are denoted by Ψ and ◯, respectively. During movement, Ψ gradually scales down the displacement vector, resulting in an interpolation between the initial and desired final postures at the output. This interpolation is the desired movement trajectory. (C) Geometric interpretation of the operations in panel B. Left: The visually referenced target and initial hand positions (*TE* and *HE*, respectively, both black) are subtracted from each other to obtain the displacement vector (*TH*, purple). Middle: The displacement vector is scaled by a factor of *ψ*(*t*) (not shown), and the result is subtracted from the target representation to obtain the desired trajectory in visual coordinates (*he*(*t*), purple). Right: The decreasing unit sigmoid *ψ*(*t*) determines the rate of advancement for the computation. Note that although the subject’s hand is shown to be coincident with its expected position, this is not a requirement of the model.

I thus pursue here the question of whether observed neural activity may be better explained within a sensorimotor model that incorporates trajectory planning (see Fig 1A). The possibility of such a model has been suggested previously, but without strong references to particular neural substrates or mechanisms [28–30]. The main argument in this paper is that PPC neurophysiology can be explained in terms of a critical component of this structure, an internal kinematic model, that is responsible for generating desired trajectories in visual coordinates. Central to this mechanism is a novel sensorimotor operation, Ψ (see Fig 1B). The computation carried out by Ψ is dynamic, and it complements the spatial operations that traditionally define the scope of sensorimotor processing. That both transformation types are needed for the expression of desired trajectories may be understood intuitively. Spatial representations and their transformations are required to constrain the desired movement to a particular region of space, and to specify its overall features. A temporal transformation Ψ is necessary to generate a specific sequence of intermediate points (i.e., a path) within this space, given the overall movement plan. With these operations, the aim of sensorimotor computation shifts from the manipulation *of* movement endpoints to an interpolation *between* them.

Below, I show that this computation can be implemented modularly, using the mechanism of basis functions. The resulting feedforward network has a number of attractive properties. Functionally heterogeneous (i.e., pure and intermediate) representations emerge naturally, and the dynamics of reference frames during a simulated sensorimotor task imitates those of cortical populations. Interestingly, the natural ordering of representations in the model is also in agreement with the observed arrangement of representations in reach related areas of PPC, to within experimental error. In summary, the computation of desired reach trajectories provides a simple and coherent explanation for the spatial, temporal, and topographical properties of neuronal populations in posterior parietal cortex.

Several implications of this model contrast it with previous views of sensorimotor processing, particularly those involving posterior parietal representations. The first is that sensorimotor computation is inherently spatiotemporal. A consequence is that the traditional experimental practice of probing sensorimotor responses under static, pre-movement, conditions must be complemented by a corresponding analysis for the movement execution period for greater insight into the underlying computations. Second, underlying the apparent functional ambiguity of parietal representations is a systematic and orderly computation. Finally, online sensorimotor activity in PPC represents the sensory predictions associated with the desired movement and not the experienced one, as is commonly assumed.

## Results

### Computational structure of the internal kinematic model

We begin with the formalism for describing actual movement. Hand trajectories during goal-directed reaching have been observed to have spatial and temporal regularities, or kinematic stereotypies [23,24]: the trajectories follow approximately straight paths from their initial points to their final points. Furthermore, the hand accelerates and decelerates in such a way that its speed during reaching exhibits a bell-shaped profile. These characteristics are captured well by a phenomenological model [25] for the online hand position ***h***(*t*):

h(t)=T−ψ(t)(T−H). (1)

Here, ***T*** is the target position, ***H*** is the initial hand position, and together these will be referred to as the parameters of the task. The use of boldface type indicates a vector. The above descriptive model was originally proposed for movements in the horizontal plane, but since recent experiments show that it also holds in 3D [31], the vectorial notation will be used here without restrictions. The scalar function *ψ*(*t*) determines the interpolation rate between movement end-points. Specific expressions for the time dependence of *ψ*(*t*) are available [25], however the important aspects for the present discussion are that at movement onset, *ψ*(*t* = 0) = 1 so that ***h***(*t*) = ***H***. At movement offset, *ψ*(*t* = 1) = 0 so that ***h***(*t*) = ***T***. In between, *ψ*(t) decreases smoothly.

Several assumptions are needed to adapt this description of actual limb movement to an internal representation of it. First, for the type of sensorimotor task modeled here, task parameters are assumed either to arrive in visual coordinates, or to have been previously converted into visual coordinates. The replacements below of ***H*** by ***HE*** (= ***H*** − ***E***) and ***T*** by ***TE*** (= ***T*** − ***E***) emphasize these assumptions. Note that this also forces the trajectory signal on the left hand side of Eq (1) to be referenced with respect to a visual frame, a result which is made explicit with the replacement of ***h***(*t*) by ***he***(*t*). Second, the computation in Eq (1) is assumed to take place in two stages,

TH=TE−HE (2)

and

he(t)=TE−ψ(t)TH. (3)

Third, these equations will be taken to be valid prior to movement onset. That is, we will assume *ψ*(*t*) = 1 for *t* < 0 (see also Fig 1C, right). Eq (1) does not allow this naturally (and does not need to) since it is a phenomenological model of reach kinematics. Here, however, the derived Eqs (2) and (3) are to be read as describing a physiological mechanism, having a physical existence. Such a mechanism would be expected to achieve a well-defined state in the moments before the onset of movement. A uniformly back-extrapolated *ψ*(*t*) is the simplest way to extend the model into this so-called delay-period, and will allow us to compare its predictions with experimental data. Under these assumptions, Eqs (2) and (3) represent the proposed computation for the internal kinematic model.

Fig 1C provides a geometric interpretation of the spatial transformations that are involved. Black arrows denote the input quantities at a given computational stage, while purple arrows denote the output. In the first stage (left), a displacement vector ***TH*** is computed from the visually referenced hand and target positions, according to Eq (2). The subsequent action of the temporal scaling Ψ is not explicitly shown, but results in the generation of *ψ*(*t*) ***TH*** (middle). This dynamically scaled displacement vector is then subtracted from the eye-centered target position to obtain ***he***(*t*), according to Eq (3).

Below, we shall implement this computation in modular form and make detailed comparisons with neural data. But, even at this level of abstraction, it is easy to see how this model accounts for several interesting features of sensorimotor representations in PPC. One feature is that because the internal kinematic model generates desired trajectories by interpolation, it requires task parameters such as the target location ***TE*** or initial hand position ***HE*** to be held in short-term memory during movement. Experiment shows that target representations are in fact sustained during movement execution in PPC [32]. Another aspect of the model is that the trajectory signal is generated directly from task parameters, without the intermediate representations of force or torque-like signals. Consistent with this requirement, posterior parietal representations are found to be best correlated with the kinematics of reaches and not their dynamics [33].

The model equations also offer insight into a peculiar aspect of sensorimotor representations in PPC. In the moments preceding movement onset, *ψ*(*t*) takes the value of unity, and Eqs (2) and (3) reduce to ***TH*** = ***TE*** − ***HE*** and ***he***(*t*) = ***TE*** − ***TH*** = ***HE***, respectively. The first of these is a sensory-to-motor transformation, converting task parameters obtained in sensory coordinates into a motor-like representation (the term “motor” here indicates a signal that is relatively closer to motor output but does not imply a movement representation at the joint-level). By contrast, the second operation reverses the first to recover the sensory quantity ***HE*** from knowledge of the motor-like signal ***TH***. Neuronal representations consistent with both of these operations are found in adjacent sensorimotor areas in PPC, but are difficult to explain using traditional models [19]. In the present view, the apparent triviality of the combined computation is simply a consequence of observing trajectory representations statically, before the onset of movement, rather than during the movement itself.

### Network expression of the internal kinematic model

A network realization of the computational model permits further comparisons with experiment. As is evident from the preceding discussion, this task requires that we express two distinct types of transformations in terms of distributed representations, spatial and temporal.

**Fig 2:**
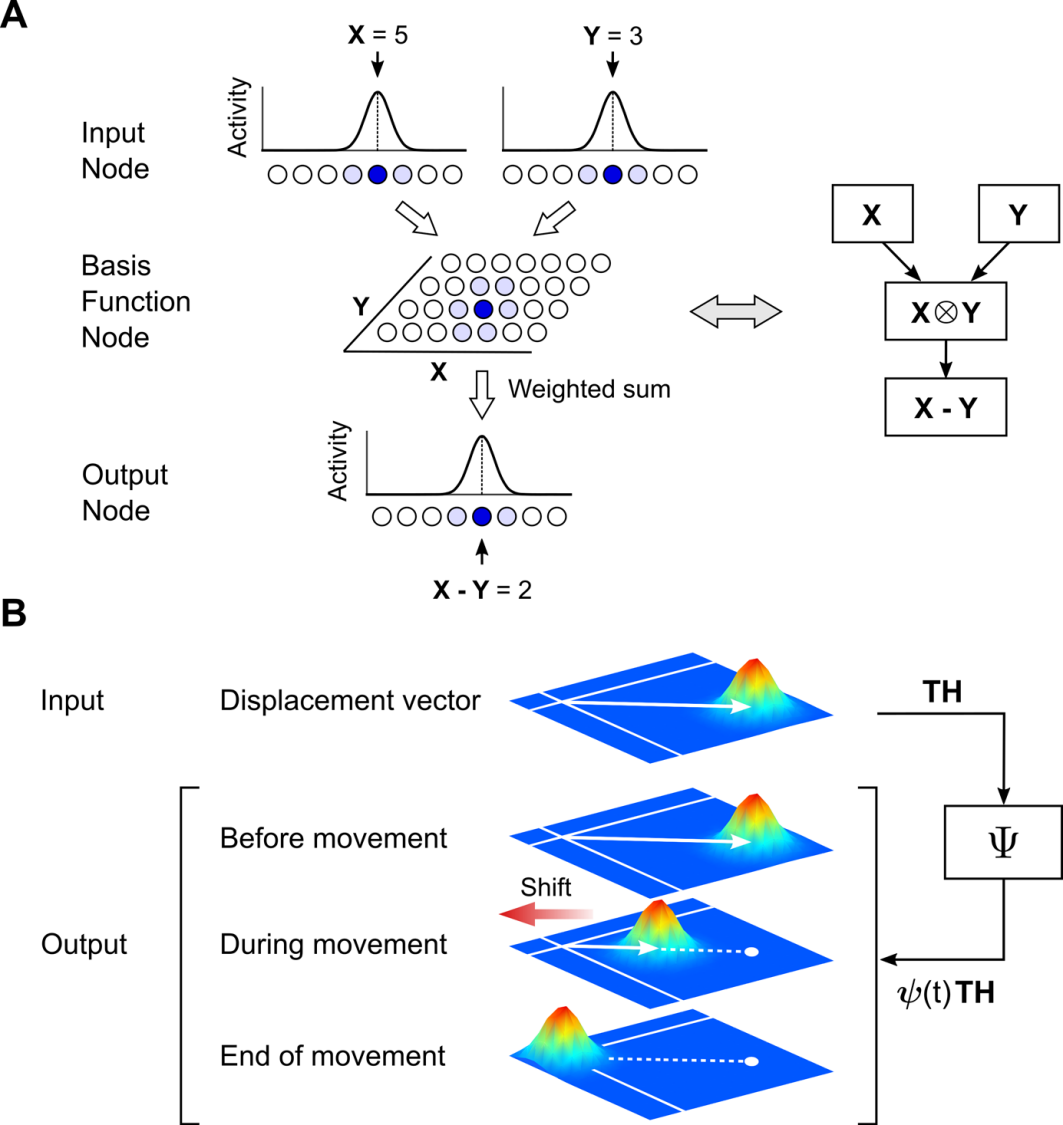
Sensorimotor operations using distributed representations. (A) Spatial operations, using basis-function networks. A feedforward, three-layer network can perform vectorial additions or subtractions, such as the operation *X* − *Y* shown here in 1D. Circles denote units within the layers (nodes), and shading intensity denotes unit activity. Units in the internal (basis-function) node have responses specific to all particular combinations of the inputs *X* and *Y*. The output representation is generated using appropriately weighted sums of internal layer activities. Right: A schematic representation of this network, using the notation ⨂. (B) Temporal operation Ψ, acting on a distributed spatial representation. Geometric interpretations of spatial quantities are shown as white arrows, and the corresponding population responses are shown as mounds of activity. White bands indicate two particular isodirections. Upper: The input activity to Ψ is a displacement vector, *TH*. Lower: Before movement onset, the output of Ψ is identical to the input provided to it. During movement execution, this activity (representing the scaled displacement vector) tends towards the null vector representation, shown at the intersection of the isodirectional bands.

The implementation of spatial transformations is the topic of numerous studies. The general strategy involves considering fully connected networks with adjustable weights, which are then trained to reproduce a given computation [9,11,13,20]. We will use basis function networks [18] for this purpose instead as they provide a considerable simplification to conventional networks while retaining their essential aspects. Fig 2A illustrates this scheme in the case of vector subtraction ***X*** − ***Y***, within a feedforward, three-layer architecture. The inputs to the network are values for ***X*** and ***Y***, which have been converted into population representations using Gaussian response functions. Projections from the input layers to the internal (basis function) layer ensure that every input combination is represented in the internal layer. In this way, there will be units that respond only or best to the input combination ***X*** = 5 and ***Y*** = 3 (shaded), for instance. Such response selectivity requires a non-linear operation, and basis function networks achieve this by combining their inputs multiplicatively [18] (to see how multiplicative responses achieve specificity, consider the a particular basis function unit with response *r* = *f*(***X*** − 5)⋅*f*(***Y*** − 3), where *f* represents a Gaussian function, and the raised dot represents multiplication. Such a unit will be inactive for all values of ***X*** and ***Y***, except in the vicinity of ***X*** = 5 and ***Y*** = 3). In the final stage, basis layer units project to the output layer, and the weights used during this stage determine the specific computation that is carried out by the network. For the example basis function unit above, a vectorial subtraction would require a mapping to the output unit representing ***X*** − ***Y*** = 2, and so on for all remaining units.

The requirements for response functions in basis function networks are less restrictive than what Fig 2A suggests. A variety of response functions can be used in addition to Gaussian shaped responses, such as sigmoidal or even semi-linear responses [18]. We will use this representational freedom in the simulations below to assign responses types to match those that are typically observed in parietal areas. For example, tuning responses will be chosen to have Gaussian shapes, while modulatory influences will take semi-linear forms (see Methods, and in particular, Eq (4)).

The generation of desired trajectories also requires a temporal operation, Ψ. Algebraically, this operation corresponds to the transformation of the displacement vector ***TH*** into *ψ*(*t*) ***TH***, as required by Eq (3). Physiological mechanisms that could achieve this are not modeled here, but Fig 2B shows the expected input–output characteristics of this transformation, assuming a topographic organization and Gaussian-like responses. The input to Ψ is a hump of activity corresponding to the displacement vector. At steady state before movement onset, this activity is repeated at the output. As the (intended) movement unfolds, the peak of the activity hump recedes from its original location towards a specific point in the organization, the null (vector) representation, at a rate resembling that of actual hand movements. The rationale for activity shifts follows the geometric interpretation of vectors, where the multiplication of a vector by a scalar produces another vector having the same orientation, but a possibly different length. For example, midway through the intended movement, *ψ*(*t*) = 1/2 and so *ψ*(*t*) ***TH*** = ***TH*** / 2. In a topographic representation that is also uniformly arranged, the output of Ψ at this instant would correspond to a locus of activity lying midway between the representations for the displacement vector and the null vector.

**Fig 3:**
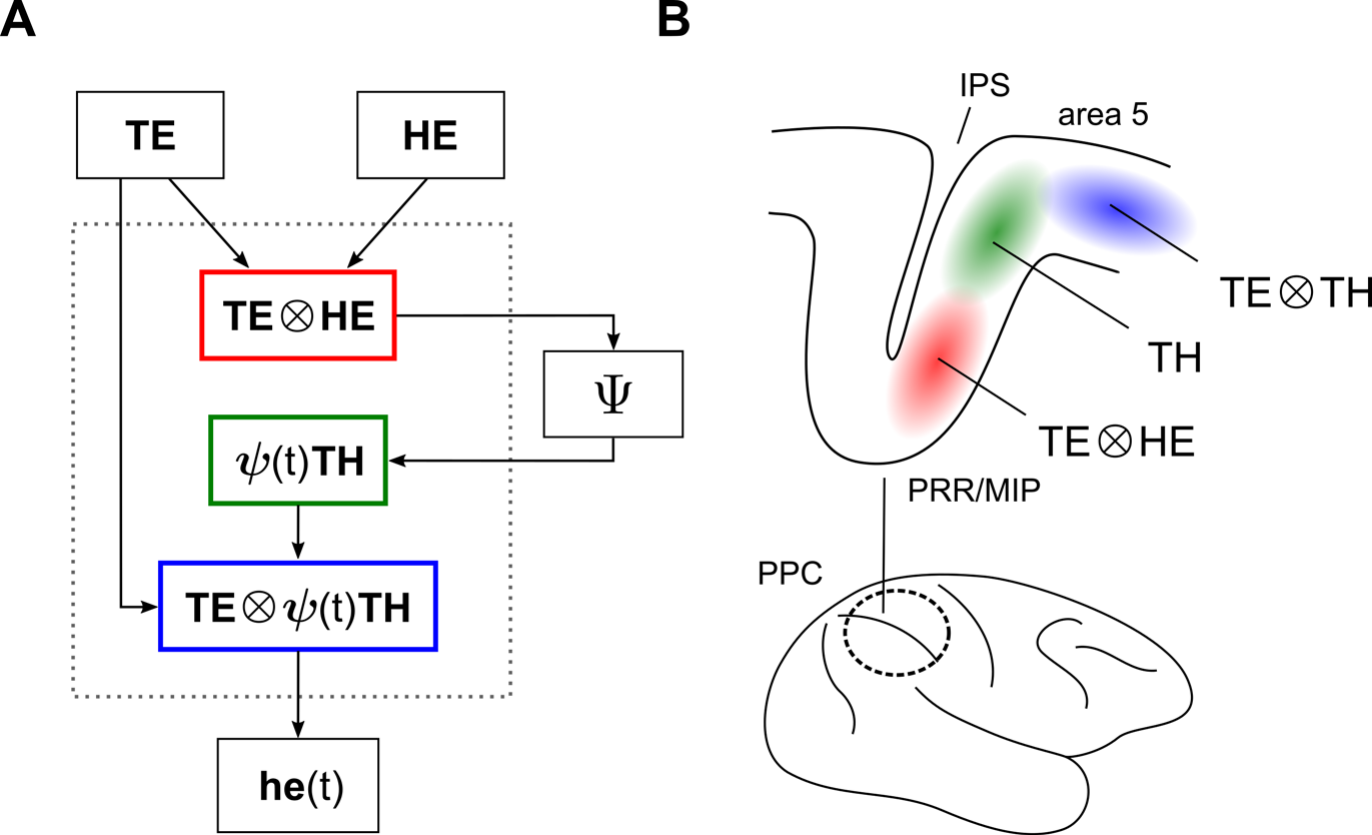
Network implementation of the internal kinematic model. (A) The computation in **Fig** 1B, carried out using schematically shown distributed representations. Basis-function nodes (involving ⨂) enable vectorial subtractions. Node Ψ is responsible for the dynamic vector scaling. Immediately before movement, *ψ*(*t*) = 1, and the internal nodes of the network represent reach targets in eye-, hand-, and eye-and-hand-centered coordinates (red, green, and blue boxes, respectively). (B) Schematic summary of sensorimotor representations in monkey posterior parietal cortex, according to refs. [11,29]. Shaded regions indicate the locally predominant spatial responses before movement onset in a 1D task, in the notation of basis functions. The color scheme is chosen to facilitate comparisons with panel A. Representations are not drawn to scale and are shown without overlap. PPC, posterior parietal cortex; IPS, intraparietal sulcus; PRR, parietal reach region; MIP, medial bank of IPS.

A schematic of the internal kinematic model, given these mechanisms, is shown in Fig 3A. The inputs (top) are distributed representations of the initial and desired final positions of the hand, in visual coordinates. The output (bottom) is the expected position of the hand during movement, also in visual coordinates. Internally, the network is feedforward in structure. A basis function node (schematically shown by the red box) first computes the displacement vector, as defined by Eq (1). The output of this stage is then relayed to Ψ, without being represented explicitly in between. Indicated here by a distinct node, Ψ dynamically modulates the displacement vector as shown in Fig 2B, and sends its output to another node where it is explicitly represented (green box). Finally, another basis function node (blue box) executes the vectorial subtraction stated in Eq (2) to produce the desired hand trajectory.

The effect of using basis functions is that the internal nodes of the network are constrained to represent task parameters in particular combinations, and in a particular order. Consider the steady state of the network in Fig 3A immediately before movement onset. During this so-called delay period, *ψ*(*t*) = 1, and the spatial characteristics of the network are obtained by omitting factors of *ψ*(*t*) from the functional descriptions of the nodes, since then *ψ*(*t*) ***TH*** = ***TH***, etc. As a result, the first basis function node (red box) will co-represent targets in a gaze-centered reference frame together with the hand position, also in gaze-centered coordinates. The second basis function node (blue box) will co-represent targets in gaze-centered coordinates together with the target in hand-centered coordinates (note that the description “target in hand coordinates” follows common usage, but should be understood to mean “target relative to hand, in eye coordinates”. Similarly, the term “displacement vector” is an abbreviation for “displacement vector in eye coordinates”). In short, the encoding of targets in the internal nodes of the network and before movement onset may be stated as being eye-, hand-, and eye-and-hand-centered.

Experiments have revealed a similar structure in monkey posterior parietal cortex, during the delay period. The spatial and anatomical characteristics of subpopulations extending from the parietal reach region (PRR) towards area 5 are succinctly put by Buneo and Andersen, who write: “In PRR, reach targets are encoded in eye coordinates while in the adjacent area 5 they are encoded in both eye and hand coordinates or exclusively in hand coordinates (in the cells closest to the cortical surface)” [29, p. 2600]. One may add to this that neuronal responses in PRR are gain modulated by hand position, referenced in eye coordinates [11]. Fig 3B provides a summary of these findings, using the notation of basis functions. A comparison of the experimental representations in this figure (as scanned from PRR to area 5) with the model representations in Fig 3A (considered during the delay period, and as read from top to bottom) shows a clear correspondence between the two. The schematic nature of Fig 3B is important to note; the shaded areas denoting distinct representations are not shown to scale. For clarity, they are also shown without overlap, even though functional representations in PPC are known to intermingle appreciably. This schematic is nevertheless useful for highlighting the similarities between the model and data, in the instants before movement onset.

This similarity also extends to a broad spatio-temporal characterization of neuronal responses in PPC, as a function of their anatomical location. Single unit recordings show that task parameters are more likely to be found in MIP/PRR, while movement related signals are more commonly found in area 5 [32,34–36]. It is evident from Fig 3A that the ordering of nodes in the model shows a comparable functional transition, as scanned from top to bottom. The eye-centered node (red) encodes a task parameter, and the static signal it represents is required to be maintained during movement. By contrast, the downstream hand- and eye-and-hand-centered nodes (green and blue, respectively) encode dynamic signals, which result in the generation of a desired reach trajectory.

### Heterogeneous spatial representations

**Fig 4:**
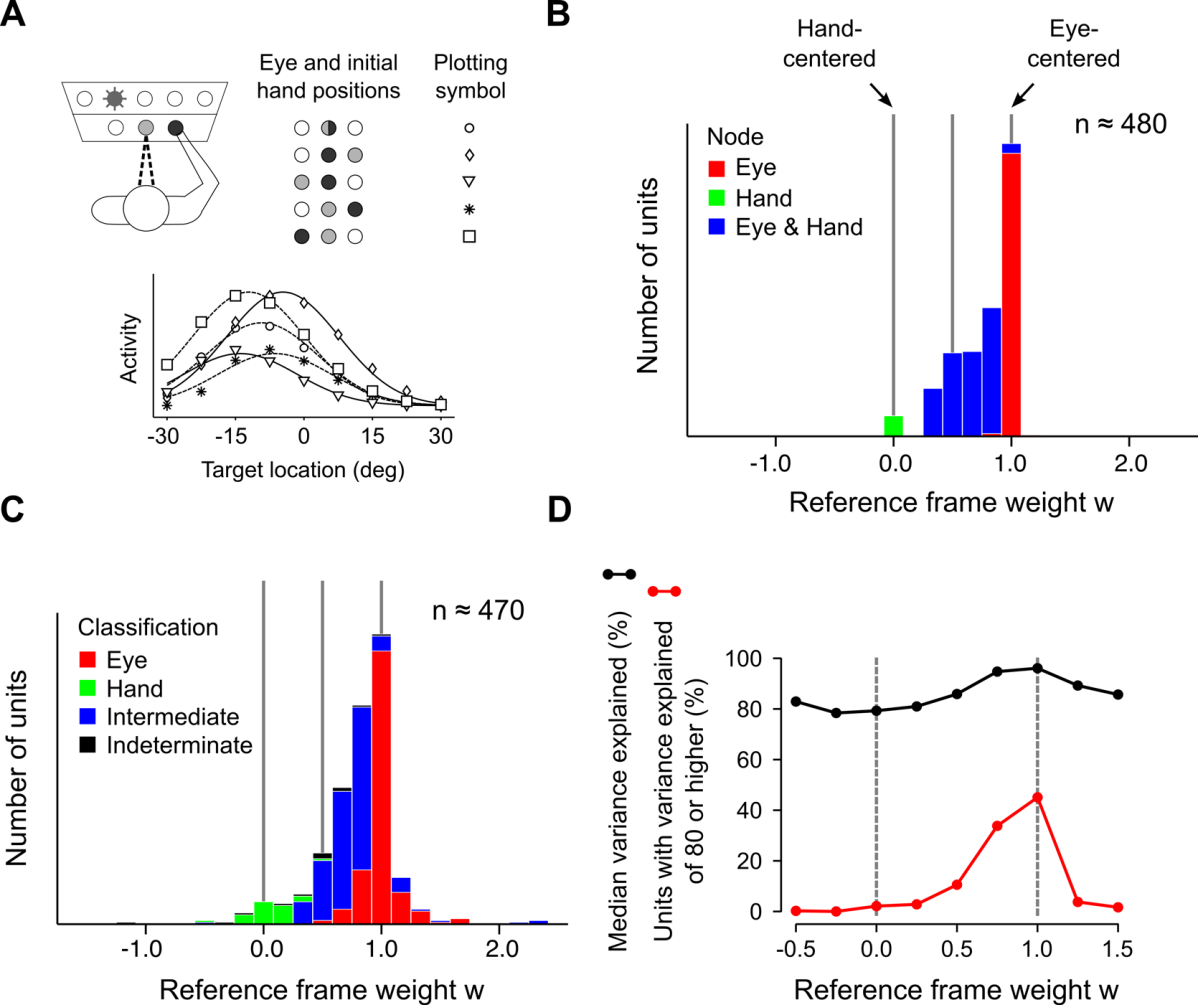
Emergence of heterogeneous spatial representations during movement planning. (A) Upper: The delay period of a simulated visuomotor task. Reaches are planned to targets (not all shown) from combinations of eye and initial hand positions, in 1D but displaced vertically for illustration purposes. Lower: The activity of a sample unit from the eye-and-hand centered representation in the network model. Regression analysis yields a reference-frame weight *w* ≈ 0.6 for this unit. (B) Distribution of reference frames for units from all internal nodes of the network model. Color-coding of the histogram is identical to Fig 3A, and indicates the node from which units originate. (C) Distribution of reference frames for noise-injected units. Color-coding is now based on the functional classification of units, using stepwise-regression (Methods). Compare with Fig 3A in ref. [2]. (D) Variance explained (*r*^2^; black curve) according to the full model (Eq (5)) by units in panel C. Data points represent the median *r*^2^ for units within bins centered about the given point. Variance explained for units satisfying *r*^2^ ≥ 0.8 (red curve). Compare with Fig 3C in ref. [2].

We are now in a position to address the perplexing issue of functional heterogeneity, from the point of view of the model. Experimentally, this heterogeneity is observed when neuronal responses from the delay-period are classified using a fitting procedure [2,4,14]. To evaluate the corresponding prediction for the model, we consider populations of units drawn from the internal nodes of the network, and simulate their responses for typical combinations of eye, initial hand, and target positions (for consistency with experimental conditions, simulations from here on are restricted to 1D, and the use of boldface type for spatial variables discontinued. See also Methods). As in the analysis of experimental data, these responses are then fit to a non-linear classification model, as shown in Fig 4A. The outcome, for each unit, is a weight parameter *w* that indicates the encoding frame for reach goals; a value at the origin denotes hand-centered encoding, whereas a value of unity denotes eye-centered encoding. The resulting distribution of reference frames is shown in panel B**.** Units from the hand-centered and eye-centered nodes acquire weights that coincide with their expected values, at *w* = 0 and *w* = 1, respectively. By contrast, weights for units from the eye-and-hand-centered node occupy the region that lies in between the pure representations.

It might be argued that experimental observations of functional intermediacy could be due to pure but noisy representations, in which case the result in Fig 4B would amount to a spurious explanation. This is unlikely, as experiment indicates. Working in reach-related areas of PPC, Chang and Snyder [2] found no significant difference in classification quality between gaze and hand-centered cells, and intermediate cells. Furthermore, their empirically determined reference-frame distribution has an internal structure that is difficult to attribute to noise. By using a stepwise regression method, these authors further classified cells as eye-centered, hand-centered, intermediate, or indeterminate, and their results are shown in Fig 3A of ref. [2] (note: intermediately classified cells in this context should not be confused with intermediate representations which are simply those from the region 0 < *w* < 1). The overlap of functional classifications in the empirical distribution is noteworthy, and shows that despite having similar reference frame weights, units in the intermediate region are not functionally homogeneous and can use different schemes for encoding information.

The internal kinematic model also explains this additional structure. Fig 4C shows the result of injecting Gaussian noise into the responses of model units, before also classifying them using stepwise regression (see Methods). In addition to a unimodal distribution peaked about the gaze-centered reference frame, the model gives rise to similarly positioned subcategories of eye- and hand-classified units, about *w* = 0 and *w* = 1, respectively, and intermediately classified units, in between (cf. Fig 3A of ref. [2]). These classifications are largely accurate: 81% of 220 units from the eye-centered node, and 94% of 17 units from the hand-centered node are appropriately classified as eye- or hand-centered. Of the 236 units from the eye-and-hand-centered node, 79% are classified as intermediate. It should be noted that although noise was introduced into the simulation for Fig 4C to make more realistic visual and regressive comparisons with the empirical data, the essential results discussed above do not depend on it.

Still, there are a few notable differences between the two sets of results. For instance, fewer cases of indeterminate classifications were encountered in the simulation, as compared to experiment. Indeterminately classified units here were typically due to the nonlinear effects of the gain modulation about its lower saturation limit. In these cases, unit responses were strongly suppressed by the modulatory response function for some postural combinations but not others, leading to partially degenerate responses and an ambiguous classification. There are also relatively fewer intermediately classified units in the experimental distribution as compared to the model. The model does not provide insight into this discrepancy, but one possibility is that an emphasis on the parietal reach region (PRR) in the experiment of ref. [2] could have led to a relative undersampling of area 5, where eye-and-hand centered representations are thought to be concentrated [19].

A final point of comparison between the model here and the data of Chang and Snyder is the fitting quality (variance-explained; *r*^2^) for units taking part in the reference frame distribution. A comparison of Fig 4D with Fig 3C of ref. [2] shows that in both cases, units are consistently well-fit by a full model (here, Eq (5)), with a slight improvement for in-bound (0 < *w* < 1) units. The latter point is further emphasized by the red curves in the two figures and shows that the distribution of variance explained for the best-fit units (here defined by *r*^2^ ≥ 0.8) are concentrated within the in-bound region. Overall, then, these results make it clear that the contact between model and experiment is not due to noise, and that the hypothesis of trajectory computation can provide a parsimonious explanation of heterogeneous reference frames in PPC.

### Switching of spatial representations

**Fig 5:**
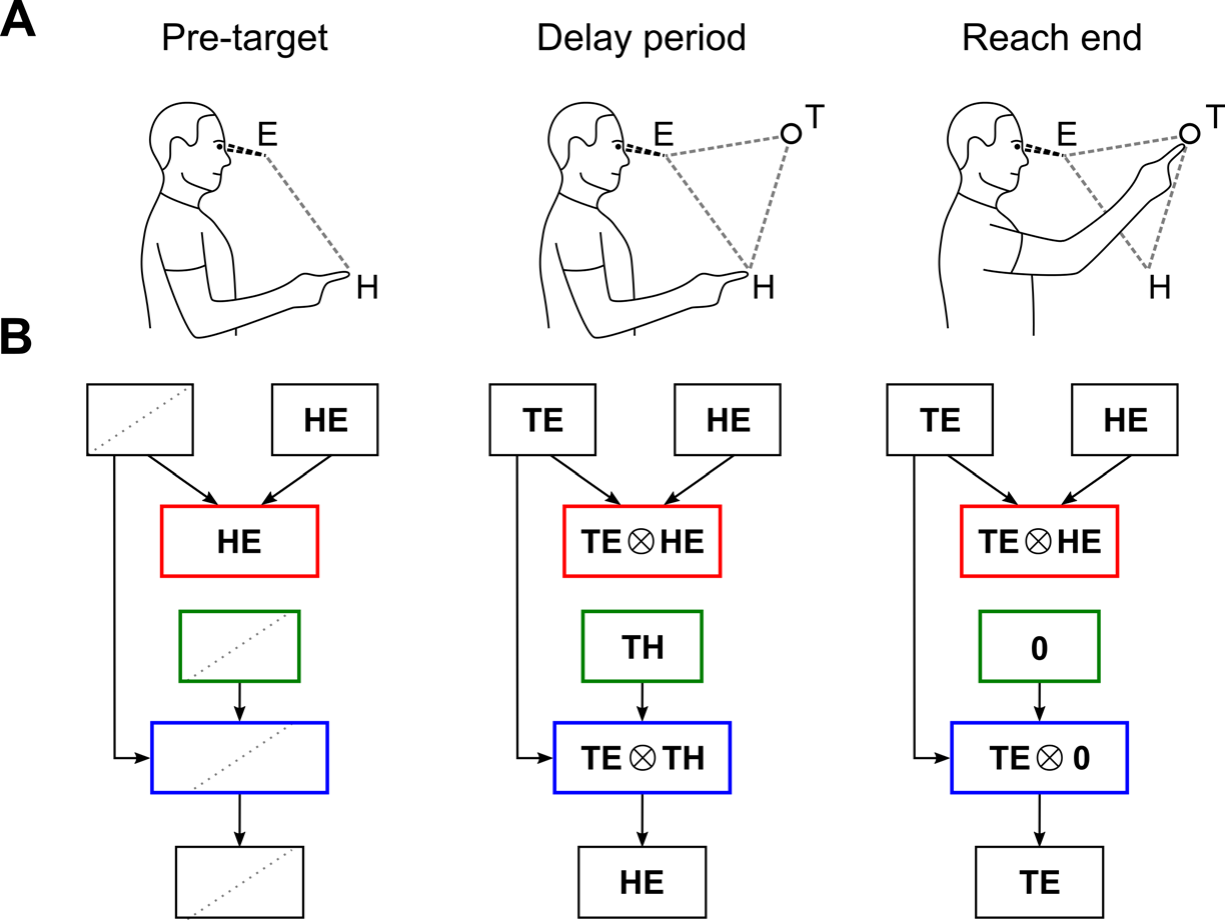
Switching of spatial representations during a visuomotor task. (A) Timeline for a typical visuomotor task. Pre-target period: the subject eye-fixates and maintains a given posture. Delay period: A reach-target is presented, but movement is withheld. End of movement: Subject acquires the target. (B) Network states during the task. The functional loop involving node Ψ is omitted for clarity. Diagonal dotted lines indicate inactive nodes, and 0 denotes a null vector representation.

Up to this point, the relationship of the internal kinematic model to empirical data was considered largely under static conditions, corresponding to the instants immediately preceding the onset of movement. We now turn our attention to the predictions of the model in the wider context of a sensorimotor task. At this point it is helpful to briefly describe the structure of a typical visuomotor experiment (see Fig 5A). Subjects begin a trial by fixating their eyes at a specified location and adopting a particular posture. A reach target is then cued but movement withheld during this delay period. Following a “go” cue, subjects are required to acquire the indicated target while maintaining visual fixation.

The representations in the network during this process will reflect the inputs to the model and its internal dynamics (Fig 5B). During the pre-target stage, only the initial hand position signal is available, and the first internal node of the network will represent this signal exclusively. Nodes downstream from this depend directly or indirectly on the availability of a target signal, and so will not be active. Following target onset and the decay of any associated transients (which are not modeled here), the network will adopt a sequence of spatial representations characteristic of delay-period activity. As we have seen above, these are the eye-centered (target) representation, the hand-centered representation, and the eye-and-hand centered representation. As the hand is transported to the target, the eye-centered representation will be maintained as required of an interpolative computation, but the hand-centered target representation will converge towards the null vector, declining steadily in strength. By contrast, the eye-and-hand representation will shift towards an eye-centered representation. At the end of the movement, only eye-centered representations will be observable.

The predicted weakening of the dynamic hand-centered representation *ψ*(*t*) ***TH*** is essential to interpreting the simulations discussed further below, and this effect may be understood from several viewpoints. At the level of computation, it is intuitively clear from Fig 1C (middle) that *ψ*(*t*) ***TH*** must vanish as the (desired) hand position approaches the target. A consequence of this is that although the behavioral parameter ***TH*** may be successfully related to (noisy) representations of *ψ*(*t*) ***TH*** before movement onset, when *ψ*(*t*) = 1, the correlation between these two will decrease progressively as the movement unfolds. At the end of the movement, *ψ*(*t*) ***TH* = 0**, and this representation will cease to co-vary with the task parameters that define movement altogether–regardless of the reach direction or extent. However, this does not mean that the associated population itself will be inactive. As the lowest graph in in Fig 2B shows, the null vector (0) encoding will then appear as a collection of behaviorally unresponsive units with a variety of baseline activities.

Several recent experiments show support for the above predictions. To see this connection, we further evaluate the dynamical predictions of the model using methodology similar to those used for empirical data.

**Fig 6:**
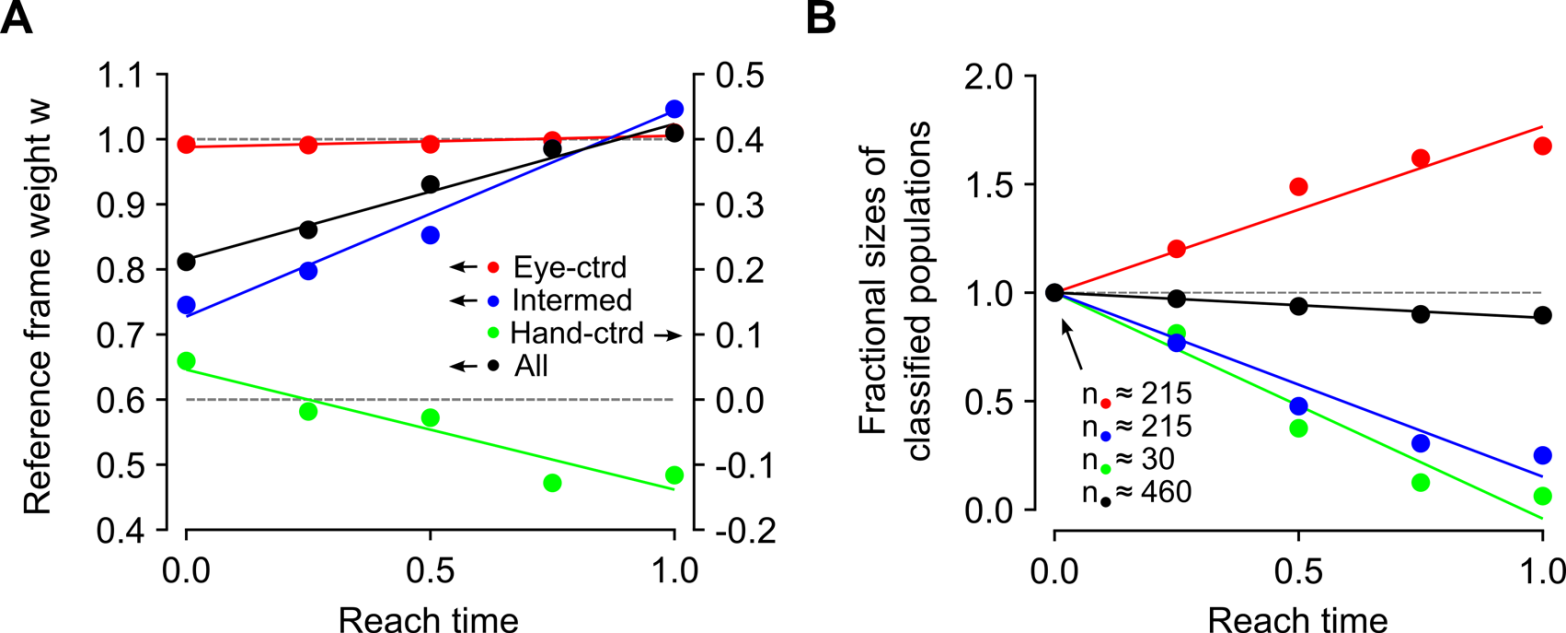
Evolution of representations during movement execution. (A) Predicted shifts in target reference frames, analyzed using regressive classification. Data points represent the mean reference frame weight for each classified subpopulation, at various points during movement execution. (B) Evolution of subpopulation sizes as a fraction of their initial values. Solid lines in both panels show linear trends, obtained using uniform weighting.

One approach is to allow the representations underlying the reference frame distribution in Fig 4C to evolve forward in time, starting from the delay period state. Fig 6A shows the resulting shifts in the encoding reference frames, broken down by classification types. The mean of the eye-classified subpopulation remains stable, at *w* = 1. By comparison, the mean of intermediately classified subpopulation tends towards *w* = 1. This shift is expected since the intermediately classified subpopulation consists largely of units from the underlying eye-and-hand representation, which according to the model, shifts to eye-centered coordinates during movement. What is surprising, however, is a drift of the hand-classified subpopulation in the opposite direction, past *w* = 0. This counterintuitive effect is an artifact of observing population dynamics using regressive methods (as would be the case in an experimental setting) and does not represent an internal contradiction within the model. Analysis shows that some of the units that are classified as hand-centered at movement onset (particularly those near *w* = 0.5) are actually misclassified units from the underlying eye-and-hand-centered node. As this underlying representation shifts towards *w* = 1 during the simulated movement, and is increasingly reclassified as eye-centered, the mean of the remaining hand-classified units drifts back towards *w* = 0. The slight overshoot to negative values of *w* is due to the increasing influence of noise in a severely weakened hand-classified population (see below). Nevertheless, the expected overall shift for the population (black) is towards an eye-centered representation.

A further prediction of the model is that the subpopulation sizes obtained by regressive analysis will also evolve, as Fig 6B shows. There are two qualitatively different mechanisms that influence this result. The hand-classified population (green) diminishes because the underlying hand-centered units become increasingly unresponsive to the task parameters at successive stages of a reach. As a result, progressively fewer of them qualify for inclusion into the distribution. By contrast, changes in the eye-centered (red) and intermediately (blue) classified subpopulations are due largely to the convergence of the underlying representations. While units from the eye-and-hand-centered representation are initially classified as intermediate, they become increasingly reclassified as eye-centric during movement. Hence, the decrease of the former subpopulation is matched by the increase of the latter. As the last two are the largest contributors to the general population, the total size of the predicted distribution (black) remains approximately constant.

Recordings in posterior parietal areas are in agreement with these predictions in general terms, although the details remain to be tested. A study by McGuire and Sabes [4] compared reference frame distributions across two behavioral epochs, the delay period and the movement period. The authors make two main observations. First, the distribution of reference frames in PPC is heterogeneous. Second, this distribution shifts towards an eye-centered representation during movement.

### Evolution of representations in area 5

Recording in the dorsal portion of area 5 (area 5d), Bremner and Andersen [16] recently characterized the evolution of reference frames of a neuronal population using the method of gradient analysis. Their work therefore presents an alternative approach to testing the dynamic predictions of the internal kinematic model.

Which network node should we use to generate predictions corresponding to this localized neuronal population? For this, we consider matching the population responses of the model and data during the delay-period. Bremner and Andersen previously found that during this behavioral epoch, their population encoded targets in a predominantly hand-centered reference frame [14]. The dynamically modulated displacement vector in the model, *ψ*(*t*) ***TH***, is the closest matching representation since during the delay period this node also encodes targets in a hand-centered reference frame, i.e., ***TH***.

**Fig 7:**
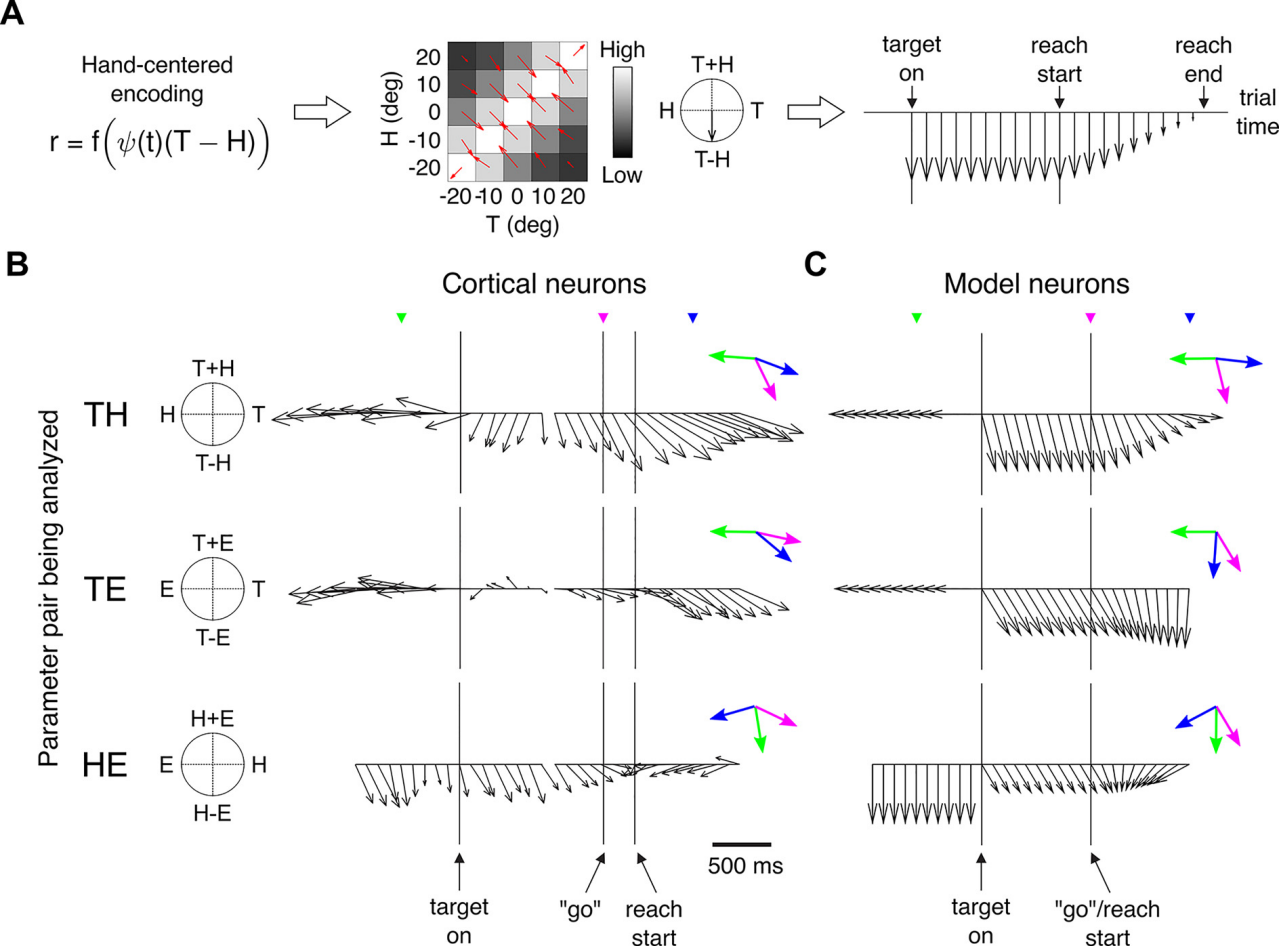
Dynamics of the hand-centered representation. (A) Gradient analysis applied to a population of units derived from the hand-centered representation in the network model. Left: The response of a particular unit from this node is determined for independent combinations of eye, hand, and target positions in 1D. Middle left: Array of responses for this unit, at a given instant and for a selected pair of analysis parameters, here *T* and *H*. The third parameter, here *E*, is held constant, at 0°. Small red arrows indicate gradients vectors. Middle right: Individual gradient vectors are used to compute an overall gradient vector for this unit. Right: The sequence of population-averaged gradient vectors illustrates the dynamics for this idealized (pure) hand-centered representation. (B) Gradient analysis for a (hybrid) hand-centered population from area 5d in posterior parietal cortex. Each row uses a different combination of analysis parameters (e.g., *T* and *H*), with the remaining parameter held constant, at 0°. Figure adapted with permission from ref. [16]. Colored arrows introduced here are gradient vectors sampled (colored triangles) 500 ms before target onset, at “go”, and at 500 ms after movement onset, and are normalized. (C) Model predictions for a similarly composed hybrid population (see text). Normalized gradient vectors (colored arrows) are sampled from the pre-target epoch, at reach onset/“go”, and at reach offset.

The application of gradient analysis to the idealized hand-centered representation from the network model is shown in Fig 7A (left, middle), for a particular instant of time. When iterated over the entire task (right), gradient analysis leads to a sequence of vectors that reveals the sensitivity of populations to task parameters. Since target-dependent representations are not available before the onset of the corresponding stimulus, gradient vectors then are uniformly zero (right). During the delay period, the sequence of gradients point in the direction of a hand-centered representation, as expected. The gradient vectors progressively shrink in magnitude during movement, as modulation depth of the underlying population tends to zero. It is instructive to compare these results with Fig 5B, which shows snapshots of the expected responses for this representation at various stages of the task.

A naive comparison of these predictions with experiment reveals a subtlety, however. Consider Fig 7B which reproduces the findings of Bremner and Andersen [16] from parietal area 5d. In particular, the top row of this panel was computed using the same analysis parameters (*T* and *H*) as was used for the simulation in Fig 7A. The differences between the two are readily apparent, even after accounting for the fact that the transition dynamics of the delay period were not modeled. On the other hand, let us remember that the hand-centered representation simulated above is an idealization, and that empirically sampled populations tend to contain a mixture of representations. In fact, the investigators estimate their nominally hand-centered representation to be composed of eye-, hand-, and eye-and-hand-centered cells in the ratio 17%: 38%: 29%, excluding indeterminate units [14]. But why should the pure hand representation in the model be sensitive to the presence of “contaminant” representations? Fig 5B reveals the reason: since the hand-centered node in the model is degenerate before target onset and at the end of movement, the response of a hybrid population based on this node will be dominated then by contributions from adjacent (i.e., eye-, and eye-and-hand-centered) nodes. For a more realistic comparison, we therefore consider a similar, but simpler, blend of eye-, hand-, and eye-and-hand-centered representations, in the ratio 25%: 50%: 25%.

Fig 7C shows the predictions for this simulated hybrid hand-centered population, which now compare favorably with the empirical results of Bremner and Andersen. Along the top rows of panels B and C, for example, gradient vectors preceding target onset point towards *H*, a representation of the hand in gaze-centered coordinates. Following the introduction of the reach target, the resultants converge, instantaneously for the model but gradually for the data, to a direction intermediate between *T* − *H* and *T*. During movement, the resultants rotate gradually towards the target representation *T*, in gaze-centered coordinates. A similar correspondence between model and data is obtained for the remaining choices of analysis parameters (rows), and for different choices of the third analysis parameter (not shown).

A concise way of viewing the model-data relationship is to display a subset of gradient vectors (colored arrows) sampled from corresponding epochs in Fig 7B and 7C. Several characteristics of the empirical data were used to determine the particular instants used for this purpose. For the pre-target epoch, the chosen time point (shown in green) is consistent with the steady state following the adoption of the initial posture, but significantly before the onset of the target stimulus. The latter condition avoids target onset effects due to the acausal binning used [16] in the smoothing of neural responses. A time point approximately 500 ms before target onset satisfies both of these constraints, but as visual inspection shows, its precise value is not critical. Similarly, gradient vectors corresponding to the movement-offset (shown in blue) are extracted from a time point approximately 500 ms after the stated onset of movement. This particular time point is also somewhat arbitrary; since limb positions were not tracked mid-flight in the above experiment [16], movement ending times for trials in the empirical data are not known precisely. However, the gradual convergence of the neural data to a steady-state during movement in Fig 7B suggests that a time point following the typical reach durations in monkeys (~300 ms; see e.g., [35,37]) and the half-width of the smoothing window (100 ms; [16]) may be suitable for representing movement offsets. The above value is taken for simplicity, and an inspection of the figure shows that this choice is also not critical. A comparison of the gradient vectors sampled from panels B and C then shows that for all combinations of analysis parameters (rows), reach onset responses (purple arrows) rotate toward the reach-offset responses (blue arrows). The rotation magnitudes are comparable, and their directions are indicative of a shift towards an eye-centered frame during movement.

### Alternative networks for the internal kinematic model

**Fig 8:**
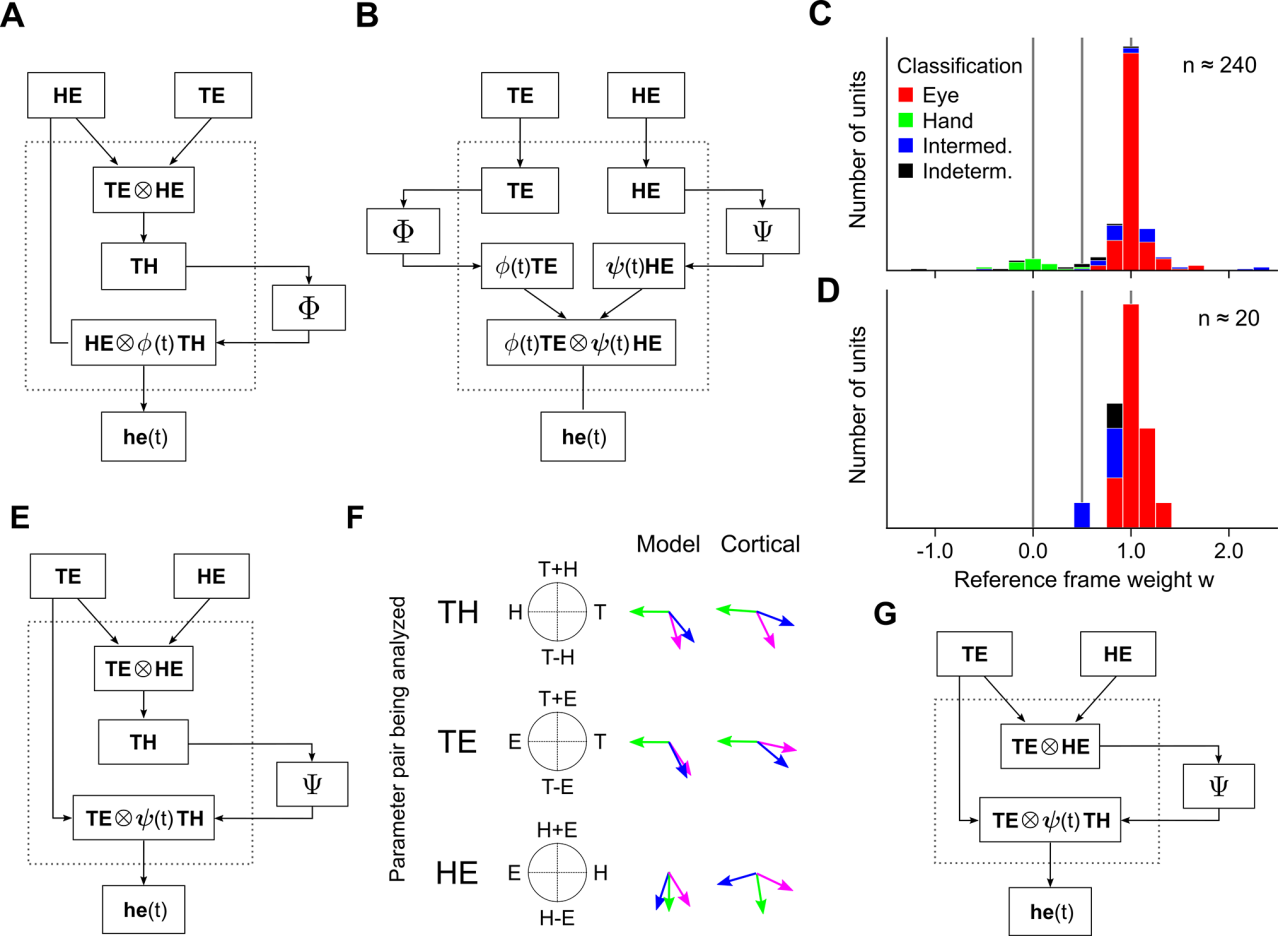
Alternative network models. Other possible implementations of the model computation (Eqs (2) and (3)), and their predictions. (A) Network using a rising unit sigmoid *ϕ*(*t*) = 1 − *ψ*(*t*). (B) Network using both rising and falling sigmoids, *ϕ*(*t*) and *ψ*(*t*). (C, D) Reference frame distributions for the models in panels A and B, respectively. (E) Network with a static hand-centered target representation. (F) Summary of gradient analysis (left) for a hybrid hand-centered representation for the network in panel E. Cortical gradients (right) are reproduced from Fig 7B. (G) A 2-layer network without an explicit hand-centered representation.

The transition from the general idea of an internal kinematic model in Fig 1A to the network model in Fig 3A involves a number of decisions. The choice of the particular form of the model Eqs is one of these. For instance, Eq (3) can be rearranged to read ***he***(*t*) = ***HE*** + *ϕ*(*t*) ***TH***, where *ϕ*(*t*) = 1 − *ψ*(*t*) is a rising unit sigmoid. A network corresponding to this rearrangement is shown in Fig 8A. It is possible to see from this figure that the delay period behavior of this model does not fully account for the empirical data–during this time, *ψ*(*t*) = 1, *ϕ*(*t*) = 0, and the representations within the internal nodes are, in order, the target in eye-centered coordinates (***TE*** ⨂ ***HE***), the target in hand-centered coordinates (***TH***), and the hand in eye-centered coordinates (since, then, ***HE*** ⨂ *ϕ*(*t*) ***TH*** = ***HE*** ⨂ **0**). This last representation differs from the experimentally observed eye-and-hand-centered signal in PPC [19]. Another way to view this discrepancy is to classify the reference frames of populations derived from the network, and the results are shown in Fig 8C. In contrast to the cortical data (Fig 3A in [2]), intermediately classified units emerging from this network (blue) do not predominate in the region 0 < *w* < 1.

Another arrangement of Eq (3) yields an expression in terms of both a rising and a falling sigmoid, ***he***(*t*) = *ψ*(*t*) ***HE*** + *ϕ*(*t*) ***TE***. A network implementation of this expression is shown in Fig 8B. This model produces neither a hand-centered target representation nor a target representation in eye-and-hand-centered coordinates during the delay period. As both of these representations are empirically observed, we conclude that such a model is also not experimentally justifiable. The above points are emphasized by the reference frame regression results in Fig 8D, which show a relative absence of intermediate units and a complete absence of hand-centered units. An interesting aspect of this particular network is that target representations, on which regressive analysis depends on, are almost entirely suppressed by the null value taken by *ϕ*(*t*) during the delay period. This explains the low numbers of units qualifying for the associated reference frame distribution.

It is hence evident that although empirically observed reference frames are a natural outcome of the desired trajectory model, they are not an inevitable consequence of it, and the particular choice of trajectory equation is important. Even then a degree of modeling flexibility remains, and the choice of which representations to show explicitly does influence the predictions of the model. For example, the model in Fig 3A does not have an explicit representation of the static displacement vector, ***TH***, but it does have a specific node for its dynamically modulated counterpart, *ψ*(*t*) ***TH***. Fig 8E shows a network that reverses this choice. While the resulting model has delay period responses identical to that of the main model (i.e., eye-, hand-, and eye-and-hand-centered tuning for *ψ*(*t*) = 1), its dynamical responses are different. Fig 8F shows the abbreviated outcome of a time-step analysis for a hybrid hand-centered representation from this network, as was considered in Fig 7C. The rotation of the purple arrows towards the blue arrows in each case indicates a shift towards an eye-centered reference frame during movement, but the magnitude of this shift is significantly reduced as compared to what is empirically observed.

Still other networks may still be generated, by varying the number of internal nodes. For example, the network in Fig 8G is again based on Eqs (2) and (3), but has only two explicitly shown internal nodes. This network is difficult to justify given the evidence for (at least) three functionally distinct reference frames predominating in adjacent patches of PPC, i.e., eye-, hand-, and eye-and-hand-centered populations.

Overall, then, we can see that the network model considered in Fig 3A is the one that satisfies the greatest number of experimental constraints. Reassuringly, the alternatives to this network model form a discrete set, and are few in number.

## Discussion

I propose here that reach related areas of posterior parietal cortex serve as a dedicated module (an “internal kinematic model”) for evaluating desired reach trajectories, in visual coordinates. This model is simple, does not have free parameters, and makes predictions that are supported by neurophysiological data. A particular strength of this model is that the empirical evidence it addresses spans multiple analysis methods and behavioral epochs: The characterization of neuronal responses according to the closest matching pure reference frames (Fig 3A), the classification of spatial responses using regressive methods (Fig 4C), and the temporal evolution of reference frames using gradient-based methods (Fig 7C).

The internal kinematic model speaks directly to several debates regarding the sensorimotor system. One issue concerns the anatomical intermingling of representations [32] and whether an expectation of topographical modularity in the nervous system is justified [14,18]. Another, more fundamental, issue concerns the functional heterogeneity of representations and the ambiguity this introduces into our understanding of sensorimotor processing [4,20]. These difficulties have led some to conclude that the underlying computation itself may not require systematic and modular processing [2]. Opponents of this view point out that there is evidence for discrete, systematic organization in the topographical arrangement of representations [14], however a coherent functional role for these has not been evident under traditional, static, frameworks [19,29,38]. The model presented here fills this need and shows that the experimental record is in fact consistent with the systematic processing of information, provided we shift our interpretation of sensorimotor computation from the manipulation of desired reach endpoints to the manipulation of desired reach trajectories.

Another debate concerns the overall transformation strategy used by the sensorimotor system. There are two competing theories regarding how reach goals might be transformed from visual coordinates into motor-like coordinates. In what may be referred to as configurational models, reach targets are transformed using positional details of all intervening parts of the body between the sensory input and motor output, such as the head, the neck, the shoulders, etc. [1,10,18,20] (here, the issue of whether these transformations are performed using a hierarchy of network layers or within a single layer is irrelevant). Alternatively, this transformation has been suggested to occur through a common, eye-centered, reference frame [19,21]. The trajectory model suggests a way out of this conflict by reinterpreting the evidence cited in support of the latter theory. In this new interpretation, the role of the common, eye-centered, representations in PPC is not to determine a competing transformation path for the target, but rather to compute the desired limb trajectory on the way to it. Because this trajectory is still referenced with respect to visual coordinates, additional operations are still needed to complete the transformation to motor-like coordinates, as shown in Fig 1A. Configurational models could be well suited for this purpose.

An internal kinematic model also compels us to reconsider the role of online activity in posterior parietal cortex. This activity is generally interpreted as representing the experienced movement, either due to the presumed role of PPC as an internal forward model (where it would serve to extrapolate the previous limb state to compensate for sensory delays) [32,39,40], or due to its presumed role in the merging of this output with incoming sensory feedback to obtain an optimal state estimate [41]. The interpretation of online posterior parietal activity using these constructions is difficult for a number of reasons. For example, a forward model explanation would require this area to process and maintain a copy of outgoing motor signals for its proper operation. Regardless of where this corollary discharge may originate from, neuronal activity in PPC activity would then be expected to correlate with force, torque, or muscle signals during movement. Yet recordings have shown that reach related activity in PPC is better correlated with the kinematics of the movement, rather than the forces required to execute it [33,42]. Furthermore, the recursive nature of forward model-like constructions implies that movement representations at each instant depend only on the information from the previous time step. The necessity of sustaining goal representations during visuomotor tasks is then not readily explainable.

The internal kinematic model takes an alternative approach that avoids these issues. Here, the online activity is obtained by generically interpolating between the given initial and desired final postures, without the continual involvement of incoming sensory feedback or outgoing motor commands. As such, this signal is independent of the actual motion of the hand, and its natural interpretation is that it represents the desired movement, or its predicted sensory consequences. Interestingly, recent experiments indicate that in many instances, it is our prior expectations about how a movement should unfold that form the basis of our motor awareness (see ref. [40] for a review). Furthermore, posterior parietal cortex in the human appears to be critical for the generation of this awareness [43]. It is tempting to speculate then that the conscious awareness of self-generated movement may have its basis in a mechanism of the type described here.

A reinterpretation of the observed lags of trajectory representations in PPC is also possible with this construction. It has been appreciated for some time that real-time kinematic representations in posterior parietal cortex are nearly simultaneous with the corresponding movement [31,36]. Because this simultaneity is incompatible with passive sensory feedback or outgoing motor commands, it has been interpreted as evidence for anticipatory mechanisms taking place within PPC [32,39]. The internal kinematic model provides an alternative account. Consider a system of motor control in which sensory feedback delays are appropriately compensated (e.g., [44]), and within which posterior parietal cortex serves only to generate desired movement trajectories. If this control system is sufficiently adaptive to permit the full attainment of desired movements, it would be expected that the overall timing of the executed movement would also approach its idealized form. The simultaneity of kinematic representations in PPC would then be due to adaptive anticipatory mechanisms associated with downstream processes, and not to PPC itself (see further below for a related prediction).

An equivalency between visual and Cartesian coordinates is an assumption made in the construction of the trajectory model, even though these two coordinate systems are known not to be identical. For example, it is known that the spherical shape of the retina introduces distortions of how we perceive the extrinsic, Cartesian world [45], yet it is not entirely clear if these distortions are compensated in PPC, if at all. On the one hand, simulations indicate that compensatory computations must occur somewhere in the nervous system if we are to successfully acquire visually determined targets [45]. On the other hand, the observed slight curvature of paths during goal directed reaching seems to suggest a lack of corrective action, at least in the planning of trajectories [26,27]. The neurophysiological experiments cited here in support of the model are not typically designed to resolve the role of PPC in this regard. The tasks used in such experiments use relatively small workspaces (typically under 40 degrees of eccentricity), and tend to constrain movements to the frontoparallel plane. By contrast, reaching errors associated with spherical projections become significant for larger displacements, and are most prominent for movements in the radial (depth) direction [45]. Differences between the planning of movements in visual and Cartesian reference frames are not clearly distinguishable under these conditions, and an equivalency between these two systems has been assumed in the interest of simplicity.

There could be alternative or complementary explanations for the evidence cited here in support of an internal kinematic model, however experiment provides constraints. For example, one interpretation of the data might be that PPC is responsible for evaluating a motor error, that is, an instantaneous vector from hand to target. Coupled with an internal forward model to compensate for sensory delays, a motor error could be part of a control policy that continuously minimizes the target-hand distance during the reach. At first sight, representations in PPC appear compatible with this hypothesis, with the presence of an eye-centered target representation in PRR and a hand-centered target representation in the adjacent area 5d. Consider, however, a simple network (Fig 2A) for carrying out the vectorial subtraction required for this transformation. This passive network would be expected to allow all signals (including visual transients) to propagate freely and immediately from input to output, and, by implication, from PRR to area 5d. However, this prediction is not supported by neural recordings [42,46], which suggest instead the presence of a gating mechanism between these two cortical areas. The empirical data reproduced in Fig 7B provides another counter example, illustrating the relatively slow emergence of the motor-like representation in area 5d following onset of the target stimulus. A motor error signal is therefore an unlikely explanation of the hand-centered target representation in PPC. More generally, this evidence argues against sensorimotor models (involving PRR and area 5d) that are entirely passive in nature.

Although the model in this paper has been discussed entirely in the context of reach related signals in parietal cortex, it is conceivable that its premise could be applicable to other sensorimotor modalities. For instance, superior colliculus (SC) in the monkey is a subcortical structure known to be critically involved in the planning and execution of saccadic eye movements [47]. Despite important differences between the ocular and skeletal motor systems, these movement modalities and their representations in SC and PPC also share a number of similarities from the point of view of a trajectory model. As with fast reaches, the kinematics of saccades is highly stereotyped [48,49]. And, as in PPC, spatial representations in SC are heterogeneous, with reference frames for targets in SC spanning the range from eye-centered to head-centered coordinates [8]. Moreover, the spatiotemporal activity predicted here for PPC has been observed in SC more directly (see [50], and especially compare Fig 2B here with Fig 2G in [51]). Given these parallels, sensorimotor representations in SC [8] could be reinterpreted in terms of a trajectory model by a remapping of the spatial variables above (i.e., replace ***E*** (eye) and ***H*** (hand) in Eqs (2) and (3) with ***H*** (head) and ***E*** (eye), respectively). The resulting model suggests that SC could be computing desired eye trajectories, in head-centered coordinates.

### Further predictions of the model and suggestions for future work

The computation of a desired reach trajectory is only useful if it is used to influence movement. So, it would be expected that the disruption of the internal kinematic model would result in difficulties in the learning of, or adherence to, stereotyped movement patterns. A recent experiment has indeed shown that bilateral lesions in area 5d of the monkey lead to hand trajectories that are curved with respect to controls [52]; however, strong conclusions cannot be drawn since this observation was not the main thrust of that experiment. A more specific study would seek evidence for adaptation deficits to visual rotations or force fields following lesions to area 5d.

An alternative to lesion studies is suggested by experiments indicating that, under certain conditions, actions and intentions can be dissociated [53,54]. Because the model here is consistent with the generation of desired movements, a prediction is that if the instantaneous hand position were decoded from a population of parietal area 5 neurons in a dissociation task, the resulting trajectory would reproduce the intended reach and not the experienced one, whereas most current models predict the reverse [32,41].

It is also possible that an experimenter could artificially activate the internal kinematic model. The network model (Fig 3A) has a simple structure in which movement parameters at the input are converted into movement representations at the output, through feedforward (but gated) computation. The mechanism is simple also because its operation does not require the merging in of external computations. A prediction, then, is that tonic stimulation of areas more closely associated with representations of task parameters, i.e., PRR, should result in decodable activity for the intended movement in area 5, where the model suggests the output of the internal kinematic model lies. It is possible to interpret clinical experiments in which patients reported motor awareness following PPC microstimulation [55] as an instance of this activation. Whether this subjective awareness of movement is also accompanied by a corresponding trajectory signal in this area is an open question.

The clear behavioral evidence pointing to the existence of a trajectory mechanism, and the parsimonious description of posterior parietal data by a corresponding network model certainly supports the notion of a temporal component to sensorimotor computation. Disappointingly however, neither the model equations nor the basis function implementation of these equations provides guidance on the essential features of a network that could help implement this operation. Even so, some general statements and rough predictions can still be made regarding its possible substrates. For instance, it is possible that synaptic dynamics within the areas that also carry out the coordinate transformations are responsible, in which case the internal kinematic model would be localized entirely within posterior parietal cortex. However, distinct areas that form functional loops with PPC could also be involved. Frontal motor areas are one such possibility [56], but an unlikely one since motor cortex in humans does not appear to be essential to PPC functionality [57]. The parietal circuit involving the brainstem and cerebellum [58,59] may be a stronger candidate. In this case, thalamic structures would be implicated, and clinical work has in fact found adaptation impairments to novel force fields following thalamic lesions [60]. It would therefore be interesting to see if regions of the thalamus that project to MIP [59] might also be encoding a dynamically modulated displacement vector.

A characteristic of this model is that it requires the maintenance of task parameters in working memory throughout the movement. Sustained representations of target locations, i.e., desired final hand positions, have already been observed, but this fact by itself could support other models of sensorimotor computation, such as the evaluation of a motor error to guide movement (but see above on why this particular model appears unlikely). The predicted continual representation of the initial hand position in a visual reference frame is a distinct, but yet to be observed, prediction of this model.

**Fig 9:**
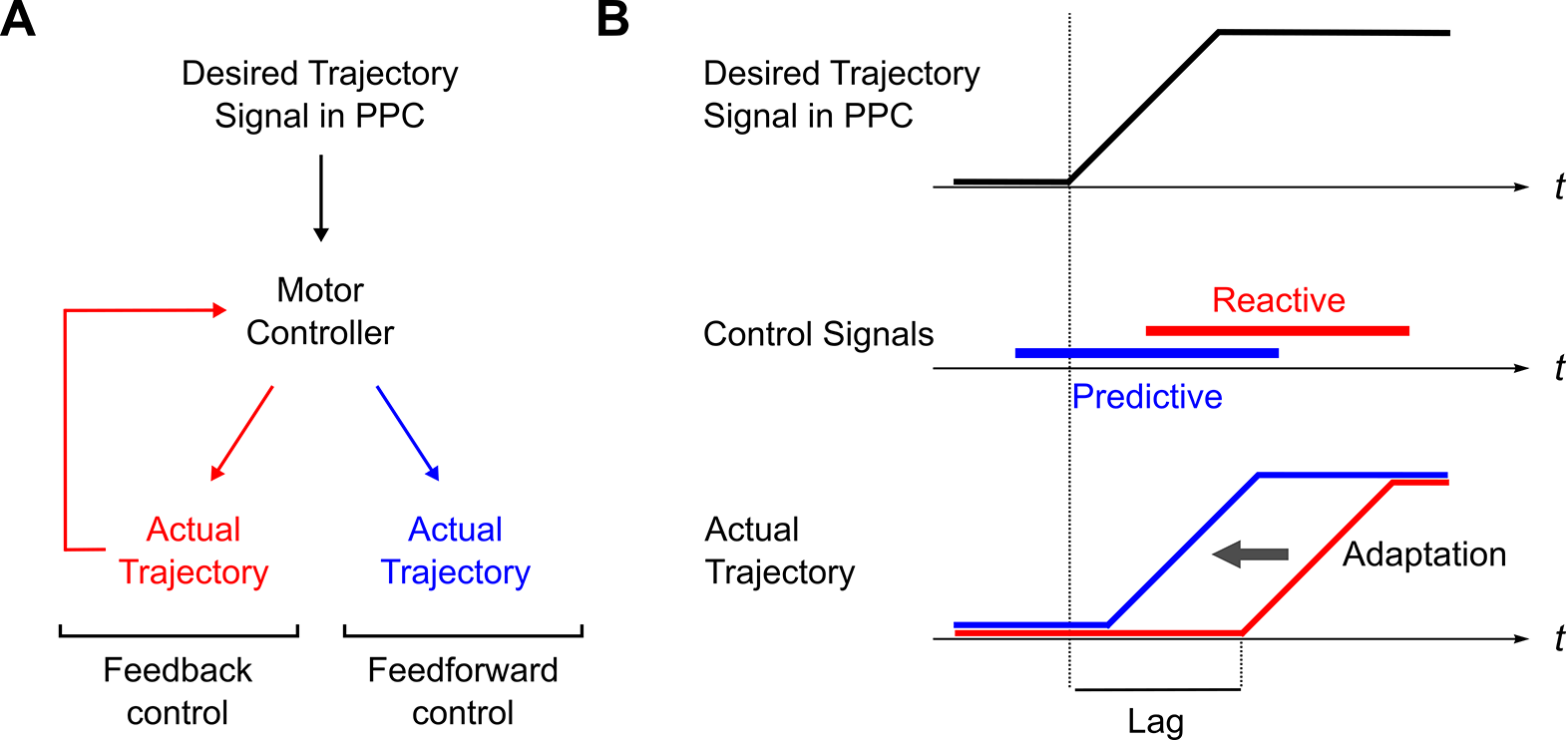
Predicted temporal shift of sensorimotor activity in PPC during visuomotor adaptation. (A) Simplified model of the motor control system. Desired trajectory signals can be used to produce movement using feedback (red) or feedforward (blue) mechanisms. During adaptation, a gradual shift from feedback (reactive) control to feedforward (predictive) control is observed. (B) When generating movement from a given desired trajectory signal (upper), the use of reactive forces (middle, red) will lead to a corresponding delay in the motor output (lower, red). As motor control forces become increasingly predictive (middle, blue), the delay between the desired and the actual movements will diminish.

A final prediction concerns the relationship between the timing of parietal representations with respect to movement. Here again, this prediction differs from that of a forward model explanation of PPC. Because the role of a forward model is to compensate for (fixed) sensory processing delays, this mechanism implies a constant temporal relationship between neural activity in PPC and limb state. By contrast, a desired trajectory model–embedded within a general framework of motor control–predicts this relationship to vary in a task dependent manner: A growing body of evidence indicates that the motor system uses two different mechanisms for producing movement, feedback and feedforward control (see, for example, [61,62], and Fig 9A). In the early stages of adaptation to novel dynamics, feedback mechanisms are used, but these are gradually replaced by feedforward mechanisms as the subject learns the appropriate dynamics. Since feedback mechanisms are reactive, and feedforward mechanisms are predictive, this shift in motor control strategy would be expected to lead to a gradual decrease in the lag time between the desired trajectory signal and the corresponding movement (Fig 9B). In other words, PPC activity during visuomotor adaptation should gradually shift from anticipating movement to being approximately synchronous with it.

## Methods

The simulations described here are based on population responses of units derived from the network model. A simplification introduced here is that although the models (computational and network) are assumed to be valid in 3D, the simulations based on them are constrained to 1D. This restriction is compatible with the typical design of sensorimotor experiments, which tend to constrain stimuli and postures to a quasi-linear arrangement within the fronto-parallel plane [11,14]. However, the above simplification goes further in that it restricts not only the task but also the encodings of units to 1D. To emphasize this point, the use of boldface symbols to denote vectors in 3D space is discontinued from here on, and in the simulation results.

The nodes in the network model of Fig 3A encod targets in eye-centered (*TE* ⨂ *HE*), hand-centered (*ψ*(*t*) *TH*), and eye-and-hand-centered (*TE* ⨂ *ψ*(*t*) *TH*) coordinates. Units within these representations were assumed to have the responses *r*_1_, *r*_2_, and *r*_3_

$$\begin{array}{c} r_{1}=A\;\text{exp}\left[-\frac{1}{2\sigma^{2}}{\left(TE-u\right)}^{2}\right]\cdot f\left[\;\pm \frac{1}{\sigma }\left(HE-v\right)\right],\\ r_{2}=A\;\text{exp}\left[-\frac{1}{2\sigma^{2}}{\left(\psi \left(t\right)\;TH-u\right)}^{2}\right],\\ r_{3}=A\exp\left[-\frac{1}{2\sigma^{2}}{\left(TE-u\right)}^{2}\right]\;\cdot f\left[\;\pm \frac{1}{\sigma }\left(\psi \left(t\right)\;TH-v\right)\right]. \end{array}$$ (4)

Here, *f*(*x*) is the semi-linear gain modulation function defined by

$$f\left(x\right)=\{\begin{array}{l} \;x,\;x\geq 0\\ \;0,\;x<0. \end{array}$$

The temporal function *ψ*(*t*) also has the piecewise linear form given by

$$\psi \left(t\right)=\{\begin{array}{l} \;1,\;-1\leq t\;\leq 0,\;\text{Delay period},\\ \;1-t,\;0<t\;\leq 1,\;\text{Movement}. \end{array}$$

More realistic forms for *f*(*x*) and *ψ*(*t*) may be used, but do not qualitatively affect the results. The notation for task parameters follows Fig 1C where *E* = eye position, *H* = initial hand position, and *T* = target position, in extrinsic coordinates. Letter pairs denote relative positions, for example, *TE* = *T* − *E*, etc. The parameter *A* = 30 determines the overall response amplitude while *σ* = 15° determines the length-scales of both the Gaussian tuning and the gain modulation. The slope of the gain modulation alternates between positive and negative values for adjacent units, a fact denoted by the ± in the argument of *f*. Noise, when modeled, was sampled from a Gaussian distribution with a mean of zero and standard deviation of 5.

Simulated populations corresponding to the internal nodes of the model in Fig 3A were obtained by considering Eq (4) for different values of the onset parameters, *u* and *v*. A set of *N* = 30 values for each onset type was generated by uniformly sampling the range −3*σ* to + 3*σ* degrees of eccentricity. This procedure resulted in a set of populations with varying numbers of units: The representation *r*_2_ is parameterized by a single onset value, *u*, and hence requires *N* units. On the other hand, the representations *r*_1_ and *r*_3_ are characterized by a pair of onset values, *u* and *v*. Since *u* and *v* both take *N* independent values, these representations result in *N* ⋅ *N* = *N*^2^ units each. Note that these populations describe the initial tiling of (visual or hand-centered) space. The question of whether a particular unit from this population is sufficiently responsive to qualify for reference frame analysis depends on the task details, and is addressed below.

### Heterogeneous reference frames (Fig 4)

Model responses were obtained for combinations of task parameters drawn from positions arranged linearly about the origin and spaced *σ*/2 apart. Targets occupied all positions in the range −2*σ* to + 2*σ*, but eye and hand positions were chosen from the restricted set shown in Fig 4A (top). This resulted in a set of 45 responses (= 9 targets × 5 eye/hand positions per target) for each model unit, at *t* = 0. These conditions represent a simplified form of the delay-period task in ref. [2].

The resulting simulated responses were analyzed using methods similar to those in single-cell studies. To prevent reference frame misclassifications, a “screening task” was first used to restrict the population to units that have a discernible tuning peak over the working range [2,38]. Responses for the condition in which *E* = *H* at a central position, with *T* at 9 locations surrounding the center, were fitted to a four-parameter Gaussian model *r* = *a* exp[−(*T* − *μ*)^2^ / 2*s*^2^] + *c*, and only units having fitted response peak locations *μ* that were at least *s*/2 within the task range were further considered. The fitting parameters *a*, *s*, *μ* and *c* were constrained to the intervals 0 to 100, 10 to 30, -45 to 45, and 0 to 10 respectively. Fitting quality was assessed using the spike-variance-explained (SVE) metric [11]. SVE takes into account both the coefficient of determination (*r*^2^) for the fit and the fitted tuning amplitude *a*, and is defined as their product. Only units having an SVE value equal to or greater than 10 were accepted.

The encoding frame for each unit was then determined by fitting its responses to a six-parameter model

$$r=a\;\text{exp}\left[-\frac{1}{2s^{2}}{\left(TX-\mu \right)}^{2}\right]\;\left(1+g\cdot HE\right)+c,$$ (5)

where *TX* is defined as *TX* = *w* ⋅ *TE* + (1 − *w*) ⋅ *TH*. Here, *w* is the reference-frame classification weight. A value of *w* = 1 indicates eye-centered encoding, and *w* = 0 indicates hand-centered encoding. The fit was performed using the *fminsearchbnd* function in Matlab (Mathworks, Natick, MA) and yielded values for *a*, *μ*, *s*, *g*, *c*, and *w*. The following constraints were imposed: 1 to 100 for *a*, 10 to 30 for *s*, −0.15 to 0.15 for *g*, −45 to 45 for *μ*, 0 to 10 for *c*, and −1.5 to 2.5 for *w*.

Stepwise regression was used to further classify populations. Denoting Eq (5) as the “full model”, two types of associated fitting models can be defined, the “hand centered” model and the “eye centered” model, obtained by fixing *w* at zero and at unity, respectively. By comparing the full model fit with its associated sub-models (F-test; *P* < 0.01), units were classified as eye-centered, hand-centered, intermediate, and indeterminate. Further details may be found in ref. [2].

### Evolution of area 5d representations (Fig 7)

A behavioral task was simulated by considering movements to independent combinations of eye, initial hand, and target positions, over the range −20° to +20° and in steps of 10°. The task comprised three distinct periods: pre-target, delay, and movement. Eq (4) determined the unit responses for the delay and movement periods. For the pre-target period (−2 ≤ *t* < −1), unit responses were obtained from Eq (4) by setting the target-related functions to unity

$$r_{1}=\text{A}\;f\;\left[\;\pm \frac{1}{\sigma }\left(HE-v\right)\right],$$

$$r_{2}=\text{A},$$

$$r_{3}=\text{A}.$$

The screening of this pre-target population was carried out based on their delay period responses, using the center-out task and SVE criterion, as above.

Gradient analysis on the remaining units was then performed for each 0.1 s time step during the simulated task as detailed previously [16,19]. Briefly, a 5-by-5 activity matrix was constructed by arranging responses for pairs of analysis parameters (i.e., *T* and *H*; *T* and *E*, *H* and *E*) while holding the third parameter in each case constant, at 0°. A gradient vector was estimated for each element of the response matrix using the Matlab function *gradient*. As a relative variable is intrinsically indistinguishable from its inverse (i.e., *TH* = *HT*) [29], the angle for each gradient vector was doubled before computing the resultant to prevent cancellations. Unit resultants were further summed to obtain the population resultant. Repeating this process for each instant over the simulated trial resulted in a progression of gradient vectors, for each node and each pair of analysis parameters. For the hybrid population results in Fig 7C, the results from the eye-, hand-, and eye-and-hand-centered representations were summed with weights 0.25, 0.50, and 0.25, respectively.

### Alternative networks (Fig 8)

The reference frame distributions in Fig 8C and 8D (corresponding to the networks in panels A and B, respectively) were obtained using methods and parameters identical those used in Fig 4. For panel C, the network responses were

$$r_{1}=A\;\text{exp}\left[-\frac{1}{2\sigma^{2}}{\left(TE-u\right)}^{2}\right]\cdot f\;\left[\;\pm \frac{1}{\sigma }\left(HE-v\right)\right],$$

$$r_{2}=A\;\text{exp}\left[-\frac{1}{2\sigma^{2}}{\left(TH-u\right)}^{2}\right],$$

$$r_{3}=A\;\text{exp}\left[-\frac{1}{2\sigma^{2}}{\left(\phi \left(t\right)\;TH-u\right)}^{2}\right]\;\cdot f\;\left[\;\pm \frac{1}{\sigma }\left(HE-v\right)\right].$$

For panel D, the internal nodes of the network in Fig 8B were simulated using

$$r_{1}=A\;\text{exp}\left[-\frac{1}{2\sigma^{2}}{\left(TE-u\right)}^{2}\right],$$

$$r_{2}=A\;\text{exp}\left[-\frac{1}{2\sigma^{2}}{\left(\phi \left(t\right)\;TE-u\right)}^{2}\right],$$

$$r_{3}=A\;\text{exp}\left[-\frac{1}{2\sigma^{2}}{\left(HE-u\right)}^{2}\right],$$

$$r_{4}=A\;\text{exp}\left[-\frac{1}{2\sigma^{2}}{\left(\psi \left(t\right)\;HE-u\right)}^{2}\right],$$

$$r_{5}=A\;\text{exp}\left[-\frac{1}{2\sigma^{2}}{\left(\phi \left(t\right)\;TE-u\right)}^{2}\right]\;\cdot f\;\left[\;\pm \frac{1}{\sigma }\left(\psi \left(t\right)\;HE-v\right)\right].$$

For the results shown on the left column in panel F, the time-step analysis follows the methods and parameters of Fig 7C. The only difference was the replacement of the dynamic hand-centered response *r*_2_ in Eq (4) by the static hand-centered representation

$$r_{2}=A\;\text{exp}\;\left[-\frac{1}{2\sigma^{2}}{\left(TH-u\right)}^{2}\right].$$
